# A deep learning approach for detecting drill bit failures from a small sound dataset

**DOI:** 10.1038/s41598-022-13237-7

**Published:** 2022-06-10

**Authors:** Thanh Tran, Nhat Truong Pham, Jan Lundgren

**Affiliations:** 1 Department of Electronics Design, Mid Sweden University, Sundsvall, Sweden; 2 Division of Computational Mechatronics, Institute for Computational Science, Ton Duc Thang University, Ho Chi Minh City, Vietnam; 3 Faculty of Electrical and Electronics Engineering, Ton Duc Thang University, Ho Chi Minh City, Vietnam

**Keywords:** Electrical and electronic engineering, Computer science, Information technology

## Abstract

Monitoring the conditions of machines is vital in the manufacturing industry. Early detection of faulty components in machines for stopping and repairing the failed components can minimize the downtime of the machine. In this article, we present a method for detecting failures in drill machines using drill sounds in Valmet AB, a company in Sundsvall, Sweden that supplies equipment and processes for the production of pulp, paper, and biofuels. The drill dataset includes two classes: anomalous sounds and normal sounds. Detecting drill failure effectively remains a challenge due to the following reasons. The waveform of drill sound is complex and short for detection. Furthermore, in realistic soundscapes, both sounds and noise exist simultaneously. Besides, the balanced dataset is small to apply state-of-the-art deep learning techniques. Due to these aforementioned difficulties, sound augmentation methods were applied to increase the number of sounds in the dataset. In this study, a convolutional neural network (CNN) was combined with a long-short-term memory (LSTM) to extract features from log-Mel spectrograms and to learn global representations of two classes. A leaky rectified linear unit (Leaky ReLU) was utilized as the activation function for the proposed CNN instead of the ReLU. Moreover, an attention mechanism was deployed at the frame level after the LSTM layer to pay attention to the anomaly in sounds. As a result, the proposed method reached an overall accuracy of 92.62% to classify two classes of machine sounds on Valmet’s dataset. In addition, an extensive experiment on another drilling dataset with short sounds yielded 97.47% accuracy. With multiple classes and long-duration sounds, an experiment utilizing the publicly available UrbanSound8K dataset obtains 91.45%. Extensive experiments on our dataset as well as publicly available datasets confirm the efficacy and robustness of our proposed method. For reproducing and deploying the proposed system, an open-source repository is publicly available at https://github.com/thanhtran1965/DrillFailureDetection_SciRep2022.

## Introduction

Drill fault detection systems are widely used in factories to prevent machine failure. The drilling machine is included 90 or 120 drill bits to drill thousand of small holes on the surface of the metal^1^. When drill bits break, there is a need for manual drilling, and post-production, which is resource-demanding and overall costly for the company. The maintenance technician stops the machine every 10 min to identify any broken drill bits and change them before re-initiating the drilling machine. Therefore, a fault detection system for the drilling machine is very crucial to minimize the downtime of the machine as well as the maintenance cost.

Many studies have been conducted on detecting and diagnosing drill failure in the past decade. Choi et al.^2^ proposed a time domain and frequency domain feature extraction method named characteristic parameters of the drill failure (CPDF). In the second step, a multilayer perceptron (MLP) was used to predict drill failure based on the drill state index threshold. This would lower the error rate. To improve the accuracy of diagnosis for drill failure, Skalle et al.^3^ proposed a method based on symptom detection (e.g., soft formation, cutting accumulation, local dog leg). Kumar et al.^4^ used vibration signals to detect and classify drill failures using three different classifier schemes: artificial neural network (ANN), support vector machines (SVM), and Bayesian classifiers. Because vibration-based signals often contain noise, several techniques were required to remove noise and separate the sources to improve fault detection accuracy.

Researchers have used sound and vibration analysis in recent years to detect and classify faults^5^. As a result of the development of deep learning, Convolutional Neural Networks (CNNs) were used to automatically extract features to diagnose and classify faults on machines, especially drill machines. Due to the advantage of acoustic analysis over vibrations, Glowacz^6^ proposed an acoustic-based fault detection method for electric impact drills and coffee grinders. These acoustic features, including the root mean square (RMS) and a method selection of amplitude using a multi-expanded filter (MSAF-17-MULTIEXPANDED-FILTER-14), were used to classify fault status by the nearest neighbor classifier. Additionally, to detect the fault in electric impact drills, it is necessary to determine the fault of the gearbox device of the drill, since the gears are the main component of the power transmission. Jing et al.^7^ proposed a method for detecting electric impact drill failure by using logistic regression from time-varying loudness and acoustic signals.

Recently, a number of techniques have been investigated in the field of fault detection and machine condition monitoring. Hou et al.^8^ used wavelet packet energy to extract features from acoustic signals, then applied a feature selection method based on the Pearson correlation coefficient to select features. The selected features were used to classify the fault status with a neural network classifier. In addition to synchronous hydraulic motors, this approach can be applied to other rotating machines as well. In another approach, Wang et al.^9^ proposed a multimodal method to detect bearing faults by fusing acoustic and vibration signals collected from the accelerometer and the microphone using the 1 dimensional CNN.

In recent years, deep learning has had a great deal of success in the detection and diagnosis of mechanical faults by using vibration and acoustics signals^10–16^. Besides, recent studies have demonstrated that image representations of sound signals can be used to train the deep learning architecture for sound classification tasks. Researchers have proposed a lot of image representations for sounds, such as Mel-frequency cepstral coefficients (MFCCs)^17,18^, spectrogram^19^, Mel spectrogram^20^. Additionally, many state-of-the-art deep learning models have been used for sound classification. Boddapati et al.^19^ compared the classification accuracy of AlexNet and GoogleLNet on three different feature representations of sound (spectrogram, MFCC, and cross recurrence plot). A variant of conditional neural networks, called masked conditional neural network (MCLNN) has been proposed by Medhat et al.^21^ for classifying sounds. Researchers have used dilated CNNs with dilated filters and leaky ReLU activation functions^17,22^. The effect of modulating the dilation rate in dilated CNN on sound classification was compared in Chen et al.^22^. Recent studies have shown that recurrent neural networks (RNN) produce excellent results for variable-length sound sequences. Wang et al.^23^ proposed a CNN architecture with a parallel temporal-spectral attention mechanism to capture certain frames where sound events occur and pay attention to varying frequency bands. Zhang et al.^24^ proposed a CNN architecture to learn spectro-temporal features and a bidirectional gated recurrent unit (Bi-GRU) with a frame-level attention mechanism for sound classification. Moreover, drilling sound analysis has been used in orthopedic surgical operations, e.g., manual drilling bone. For instance, Torun and Pazarci^25^ proposed an ANN-based classifier scheme to classify whether breakthrough or non-breakthrough occurred, using parametric-based Power Spectral Density Estimation. Seibold et al.^26^ fed log-Mel spectrograms of drilling sounds into ResNet-18 to detect drill breakthrough events and demonstrate the potential of deep learning-based acoustic sensing for surgical error prevention.

Our article proposed an approach to detect drill machine failures based on drill sounds from Valmet AB. This is a company in Sundsvall that provides processes and equipment for biofuels production. Valmet AB is currently operating multiple drilling machines to drill holes in metal materials. Most drilling fault detection studies, however, used a large, balanced dataset. Broken drill bits do not occur quite often, hence the sound of broken drill bits only accounts for a small percentage of the total. It is difficult to train advanced deep learning models on small datasets in real-world applications. In addition, the extracted features from raw sound signals are insufficient for classification because the sample duration for sounds in the dataset is around 20.83 ms and 41.67 ms. This makes it more challenging to compare our results to those of previous research in the field of drilling sounds classification. As a result, an end-to-end deep learning system faces many challenges when it comes to detecting drill faults. To overcome these difficulties, data augmentation methods were applied to generate more samples of the dataset. The augmentation methods were shifting the sound by 5 ms and increasing the volume by 2. These sounds in the augmented dataset were converted into log-Mel spectrograms. In addition, a CNN combined with an attention-based LSTM was proposed for classifying drill sounds. Feature maps were extracted from the log-Mel spectrograms using CNN, and then an LSTM layer was used to learn high-level global feature representation from extracted features. Leaky ReLU was used in CNN instead of ReLU to alleviate the potential problem that CNN stops learning when the ReLU has a value of less than zero. Leaky ReLU helps CNN continue learning when input values are negative. To focus on the important parts of drill sounds and discard the unnecessary parts, an attention layer was added after the LSTM.

## Proposed methodology

**Figure 1:**
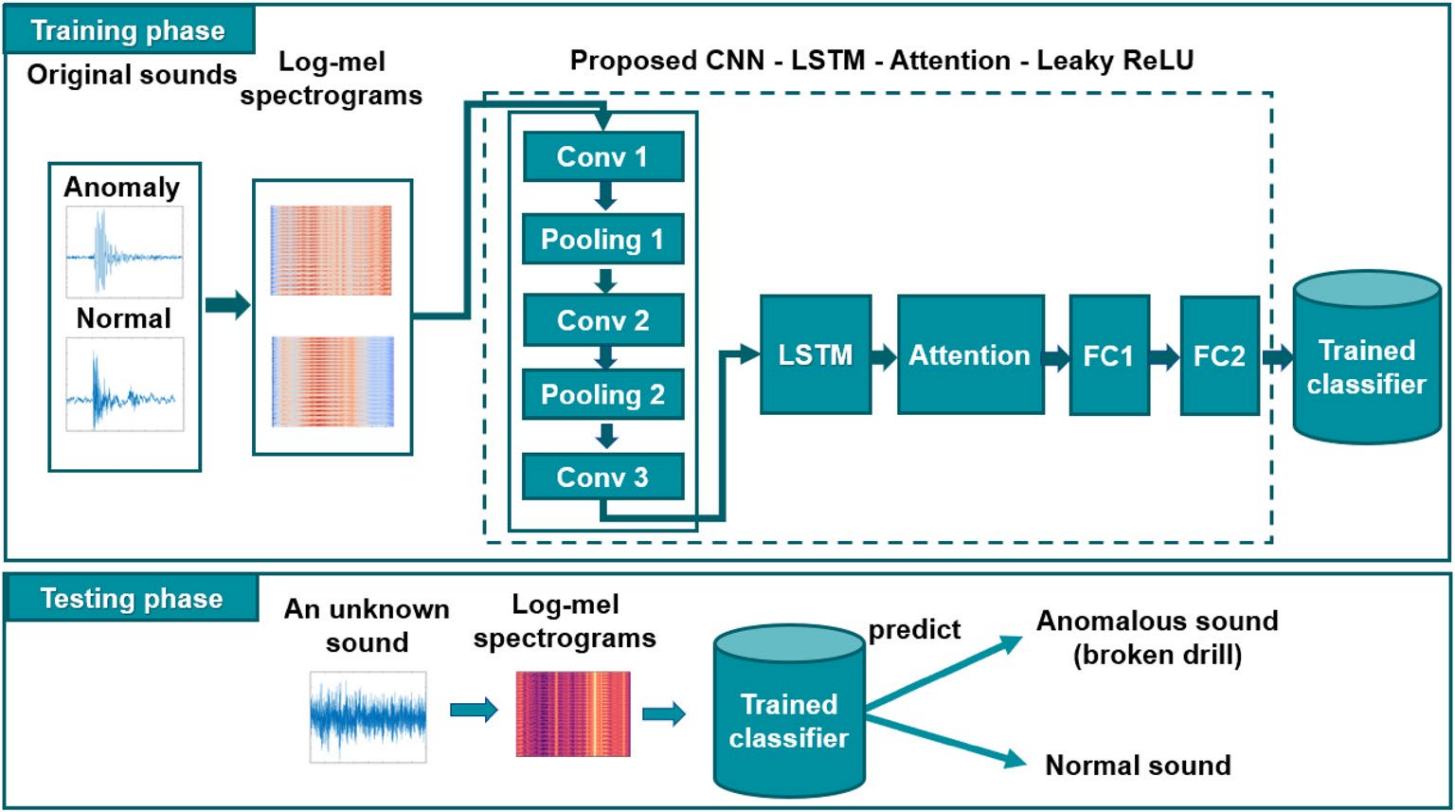
The proposed methodology.

The proposed architecture is described as shown in Fig. 1. Initially, audio augmentation methods were applied to original sounds to increase the number of samples in the dataset. In the next step, a small CNN architecture that includes five layers was proposed to generate features from the Mel spectrograms of sounds. Finally, these features were used as the input of the LSTM with the attention mechanism to learn high-level feature representation. The details of the layers in our proposed model are described in Table 1, where *nC* is the number of classes and $(S=1)$ is the stride of 1 for the convolutional layer.

**Table 1:** The layers of the proposed model.

Layer	Kernel/size	Output shape
Input	–	100 × 96 × 1
Conv 1 (S = 1)	3 × 3	100 × 96 × 128
Max_pooling 1	2 × 4	50 × 24 × 128
Conv 2 (S = 1)	3 × 3	50 × 24 × 128
Max_pooling 2	2 × 4	25 × 6 × 128
Conv 3 (S = 1)	3 × 3	25 × 6 × 256
Reshape	–	150 × 256
LSTM	256	–
Attention	–	–
FC 1	–	64
FC 2	–	nC

### Data augmentation

**Figure 2:**
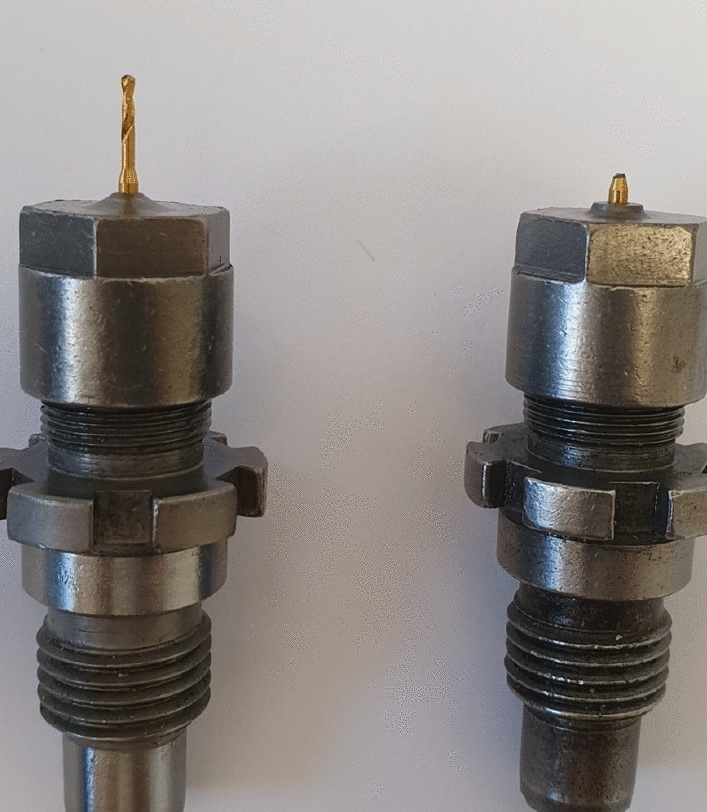
A healthy drill bit (on the left side) and a broken drill bit (on the right side)^1^.

Valmet AB drills small holes in metal plates with multiple machines. There are two types of drilling machines in a factory that are 90 and 120 bits. Figure 2 shows a healthy drill bit and a broken drill bit^1^. In this dataset, sound from a drill machine in Sundsvall, Sweden was recorded with four AudioBox iTwo Studio microphones. For capturing drill sounds, 96 kHz was used as the sampling rate. The dataset contains 134 sounds with lengths of 20.83ms and 41.67ms in two classes (normal and anomalous).

Although hyper-parameters of the model were fine-tuned to adapt to the drill fault detection task, however, the lack of sounds when the drills were broken is still a big challenge. To tackle this challenge, data augmentation methods were applied to the original sounds. Thus, the number of sounds in the dataset increased. Besides, data augmentation helps improve the generalization capability of the proposed model. There are many methods of audio augmentation such as time-stretching, pitch-shifting, volume control, noise addition, etc. It is not appropriate to apply some augmentation methods to the sounds in the dataset since they are very short at only 20.83 ms or 41.67 ms. Experimentation revealed that only time-shifting and volume control data augmentation methods are effective for the dataset.

In this article, time-shifting and volume control were applied to generate syntactical sounds. We did not add noise to the sound as an augmentation method because the sound in our dataset is very short. Noise makes it difficult to classify sounds. MATLAB provides a simple function, *audioDataAugmenter*, to augment the sound. It would be prudent to investigate other augmentation methods when applying the proposed method to other datasets.

#### Time-shifting

**Figure 3:**
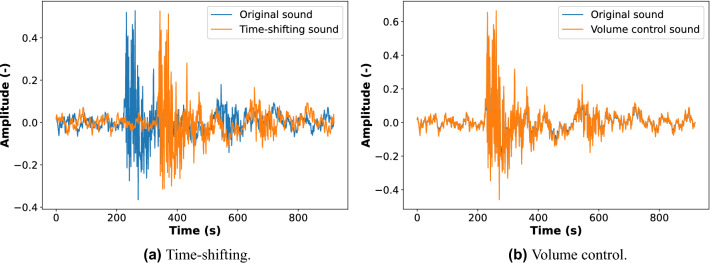
The time representation of the original fault sound and the augmented sound.

A time-shifting is the process of shifting the sound backward or forward at random. The starting point of the sound was shifted by 5 ms to the right, then padded it back to its original length. Figure 3a shows the time representation of the original fault sound and augmented sound using time-shifting.

#### Volume control

The volume was increased by multiplying the audio by a random amplitude factor. The volume gain was set as 2 dB. Using this technique, we can gain some in-variance concerning the audio input gain. The time representation of the original fault sound and augmented sound using the volume control are shown in Fig. 3b.

### Convert sounds into log-mel spectrograms

**Figure 4:**
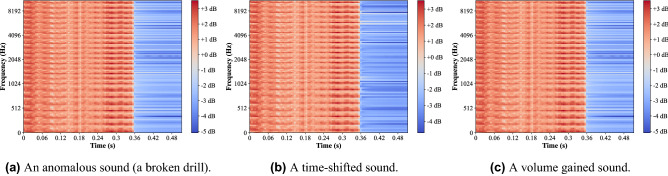
Log-Mel spectrograms of an original anomalous sound, the augmented sounds using time-shifting and volume control.

Recent advances in the field of image classification using CNN for multiple classes with high accuracy motivated us to investigate the ability of image representation of sounds to detect drill failures. In this paper, drill sounds were converted into log-Mel spectrograms to feed into the proposed CNN. The log-Mel spectrogram was generated as follows. Given a raw drill sound, the Mel spectrogram was computed using short-time Fourier transform (STFT) with Hamming windows of 100 ms and the hop length of 50 ms, the length of FFT was 2048, the sampling rate was 96 kHz, and the number of Mel-filter bank was 96. Since the authors in^27^ found that the log-scaled Mel spectrogram improves the classification accuracy compared to the Mel spectrogram. Therefore, the logarithm of the Mel spectrogram was taken as the input of the proposed CNN architecture. Figure 4 shows log-Mel spectrograms of an original anomalous sound and its augmented sound using volume control and time-shifting.

### Extract features using CNN with leaky ReLU

A CNN architecture was proposed for extracting features from log-Mel spectrograms. As a result, the third convolutional layer was used for extracting features instead of adding a dense layer at the end. Additionally, Leaky ReLU was used as the activation function. The experiment results show that using Leaky ReLU can improve the classification accuracy of the dataset. To learn global high-level feature representation, the extracted features were fed into LSTM with an attention mechanism.

The proposed CNN architecture consisted of three convolutional layers and two max-pooling layers, and six batch normalization layers with the Leaky ReLU activation functions. Log-Mel spectrograms were fed into the proposed CNN to extract high-level features for the classification task. Firstly, three convolutional layers with 3 × 3 filter kernel sizes were utilized. Three convolutional layers have 128, 128, and 256 feature maps, respectively. Secondly, a max-pooling layer with 2 × 4 filter kernel sizes were added after the first two convolutional layers. A pair of batch normalization (BN) layers with Leaky ReLU were added before and after the convolutional layers to normalize the features and reduce over-fitting.

The equation for ReLU is $$ f(x) = max(0,x)$$. When the input of the layer is negative, the ReLU is equal to zero. Consequently, gradient descents reach the value of zero and cannot converge to the local minimum. For Leaky ReLU, there is always a small slope to allow the weight update of the accumulated gradient. Therefore, although ReLU can compute faster, Leaky ReLU was used instead of the ReLU so that the layers did not stop learning when the slope of the ReLU is zero. The Leaky ReLU activation function^28^ is described by the Eq. (1):

1 $$\begin{aligned} f(x)= {\left\{ \begin{array}{ll} x, &{} \text {if}\ x>0 \\ \alpha x, &{} \text {otherwise}, \end{array}\right. } \end{aligned}$$

where $\alpha$ was set to 0.3 in this research.

### Global feature learning using LSTM and attention mechanism

In this article, LSTM^29^ was utilized to learn sequential feature maps that are extracted from the proposed CNN. The LSTM unit can be updated as in Eqs. (2)–(7):

2 $$\begin{aligned}&f_{t} = \sigma \big (W_{f}X_{t} + U_{f}h_{t-1} + b_{f}\big ), \end{aligned}$$

3 $$\begin{aligned}&i_{t} = \sigma \big (W_{i}X_{t} + U_{i}h_{t-1} + b_{i}\big ), \end{aligned}$$

4 $$\begin{aligned}&o_{t} = \sigma \big (W_{o}X_{t} + U_{o}h_{t-1} + b_{o}\big ), \end{aligned}$$

5 $$\begin{aligned}&\tilde{c}_{t} = \tau \big (W_{c}X_{t} + U_{c}h_{t-1} + b_{c}\big ), \end{aligned}$$

6 $$\begin{aligned}&c_{t} = f_{t}\odot \tilde{c}_{t-1} + i_{t} \odot \tilde{c}_{t}, \end{aligned}$$

7 $$\begin{aligned}&h_{t} = o_{t} \odot \tau (c_{t}), \end{aligned}$$

where $X_{t}$ is the mini-batch input; $i_{t}$ is the input gate; $f_{t}$ is the forget gate; $o_{t}$ is the output gate; $\tilde{c}_{t}$ is the input cell; $c_{t}$ is the cell state; $h_{t}$ is the hidden state; $\sigma$ is the *sigmoid* function; $\tau$ is the *tanh* function; *W*, *U* are the weight matrices; *b* is the bias parameter; *t* is the time step.

Due to different frame-level features contributing unequally to classifying event sound classes, an attention mechanism^30^ has been widely used in the sequence-to-sequence model. In this paper, a feed-forward attention layer^31^ was added after LSTM to specific points in a sequence when computing its output. Additionally, during the transition from the normal state of the drill bit to the broken state, the pitch of the audio changes. Therefore, the features extracted from the log-Mel spectrogram right at the moment the drill bit cracks will have an abnormality. The purpose of the attention layer is to focus on that anomaly. For the LSTM, the output of attention *att* can be defined as below:

8 $$\begin{aligned} att = \sum _{t=1}^{T}\alpha _{t}h_{t}, \end{aligned}$$

where $h_{t}$ denotes the $t_{th}$ hidden output from the LSTM at time step *t*, *T* represents the total number of time steps in the input sequence, and the $\alpha _{t}$ is the attention weight can be computed as follows:

9 $$\begin{aligned} \alpha _{t} = \frac{\exp {\big (W \cdot h_{t}\big )}}{\sum _{k=1}^{T} \exp {\big (W \cdot h_{t}\big )}}. \end{aligned}$$

## Experimental setup

The proposed method was evaluated on our Valmet’s dataset. Besides, our proposed method was also verified on a drilling dataset in^26^ called Seibold’s dataset, and a benchmark dataset, namely UrbanSound8K^32^.

### Datasets

#### Valmet’s dataset

Valmet’s drilling dataset includes 134 sounds, divided into two categories: anomaly sounds and normal sounds. After applying time-shifting and volume control augmentation methods to 134 original sounds from two categories, the extended dataset includes 402 sounds. These sounds in the augmented dataset were converted into log-Mel spectrograms to train an end-to-end model. Around 70% of the dataset (280 log-Mel spectrograms) and 30% (122 log-Mel spectrograms) were used for training and testing, respectively. When training the model on the training set, 280 sounds were divided by the ratio of 70/30 for training and validation sets.

#### Seibold’s dataset^26^

Our proposed method is also evaluated on the drilling dataset in^26^. It consists of two classes, cortical and breakthrough. In this dataset, samples were recorded at a sample rate of 44.1 kHz and a bit depth of 24 bits. Sounds in this dataset are short, just like those in our Valmet’s dataset. There are 126 sounds in the cortical category and 136 sounds in the breakthrough category. In the dataset, the sounds have varying lengths but are generally shorter than one second. The breakthrough events last between 100 and 250 ms, which is shorter than sounds in the cortical category. Our proposed data augmentation approach was not applied to this dataset. The dataset is divided into 70% (88 cortical sounds and 95 breakthrough sounds) for training and 30% (38 cortical sounds and 41 breakthrough sounds) for testing. All other experiment setups are the same as those we conducted on Valmet’s dataset.

#### UrbanSound8K^32^

UrbanSound8K^32^ was used to test the proposed method’s effectiveness in multiple classes classification with longer sounds (under or equal to 4 seconds). There are 8732 sounds in this dataset representing urban sounds from 10 classes: air conditioner, car horn, children playing, dog bark, drilling, engine idling, gunshot, jackhammer, siren, and street music. The same experimental setups were used to train 6111 sounds (70% of the dataset) and test 2621 sounds (30%). Data augmentation methods were not applied on this dataset.

### Hyper-parameters and training setup

The proposed deep learning model was trained on Intel CORE i5 8th Gen with NVIDIA Graphics Card 1050Ti. Keras library^33^ with TensorFlow toolkit^34^ that are popular deep learning frameworks were used to implement and deploy the proposed method. Additionally, the Librosa library^35^ was used to generate log-Mel spectrograms from original drill sounds.

For hyper-parameters optimization, the Adam optimizer^36^ was used with a learning rate of 0.001, a batch size of 4, a momentum of 0.9, and 100 epochs. During training, categorical cross-entropy was used as the loss function $L_{f}$ to update the network weights. It is defined as follows:

10 $$\begin{aligned} L_{f} = - \sum _{n=1}^{nC}y_{n} \log \big (\hat{y}_{n}\big ), \end{aligned}$$

where *nC* is the number of classes, $y_{n}$ is the ground truth, and $\hat{y}_{n}$ is the predicted class probabilities for the $n_{th}$ element of model predictions. Furthermore, to avoid over-fitting and to improve the generalized model, early stopping was applied to train the network with the patience of 5.

## Experimental results

Table 2 shows the results of our method on the Valmet’s dataset and the above-mentioned datasets. Our proposed method reached the accuracy of 92.62% and 97.47% on Valmet’s dataset and Seibold’s dataset, respectively. The proposed method not only performs well on small datasets with short sounds (Valmet’s and Seibold’s datasets), but it also obtains a high accuracy on the ten-class UrbanSound8K dataset with longer sounds. From the last column of Table 2, we can see that our model performs the best on the UrbanSound8K, compared to state-of-the-art methods. The performance of our proposed method reached 91.45%, slightly higher than other methods.

**Table 2:** Accuracy of different methods on Valmet’s dataset, Seibold’s dataset, and UrbanSound8K.

Methods	Datasets		
	Valmet AB	Seibold^26^	UrbanSound8K^32^
The deep residual network (ResNet)^26^	–	91.90%	–
Stride-DS-24^37^	–	–	70.90%
1D CNN^38^	–	–	89.00%
AudioCLIP^39^	–	–	90.07%
Ours	92.62%	97.47%	91.45%

### Results on Valmet’s dataset

**Figure 5:**
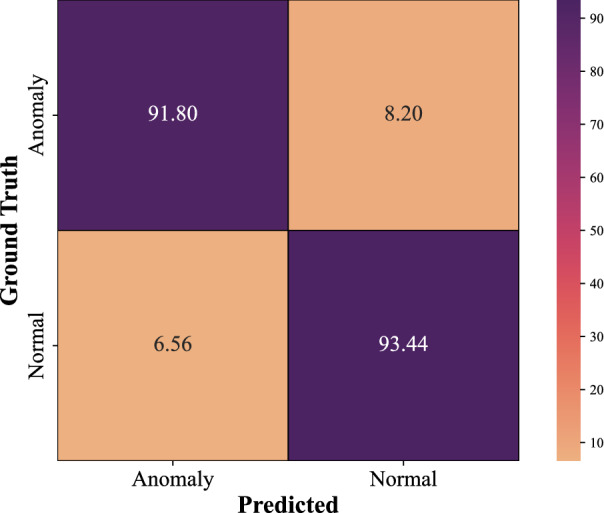
The confusion matrix for the proposed model (CNN-LSTM-Attention-Leaky ReLU) on the augmented dataset.

As shown in Table 3, the overall accuracy of the proposed method, CNN using the Leaky ReLU activation function combined with attention-based LSTM (CNN-LSTM-Attention-Leaky ReLU), was 92.62%. The confusion matrix for the proposed method is in Fig. 5. Table 3 shows the F1-score, precision, and recall for each class in the augmented dataset.

**Table 3:** CNN with Leaky ReLU in conjunction with attention-based LSTM.

	Precision	Recall	F1-score	No.
Anomaly	0.93	0.92	0.93	61
Normal	0.92	0.93	0.93	61
Accuracy			0.93	122
Macro avg	0.93	0.93	0.93	122
Weighted avg	0.93	0.93	0.93	122

No. indicates the number of samples.

#### Ablation studies on Valmet’s dataset

The role of the various modules is investigated through ablation experiments using our proposed method. As previously stated, our model has four key components: the CNN module, the LSTM layer, the attention mechanism, and the Leaky ReLU activation function. We analyze each component’s role as we eliminate modules one by one in our ablation experiments or change the activation function. The mean accuracy of all the experiments is shown in Table 4 for comparison. Using CNN with Leaky ReLU activation function in conjunction with attention-based LSTM achieves the highest accuracy of 92.62%. This result demonstrates that the Leaky ReLU and the attention mechanism can affect the overall accuracy of the proposed method when combined with CNN and LSTM. The following experiments were conducted to validate the effectiveness of our proposed method:

**Table 4:** The comparison of different models.

Model	Mean accuracy (%)
CNN-Leaky ReLU	86.89
CNN-LSTM-Leaky ReLU	90.16
CNN-LSTM-Attention-Leaky ReLU	92.62
CNN-LSTM-Attention-ReLU	91.80

##### CNN-Leaky ReLU

**Figure 6:**
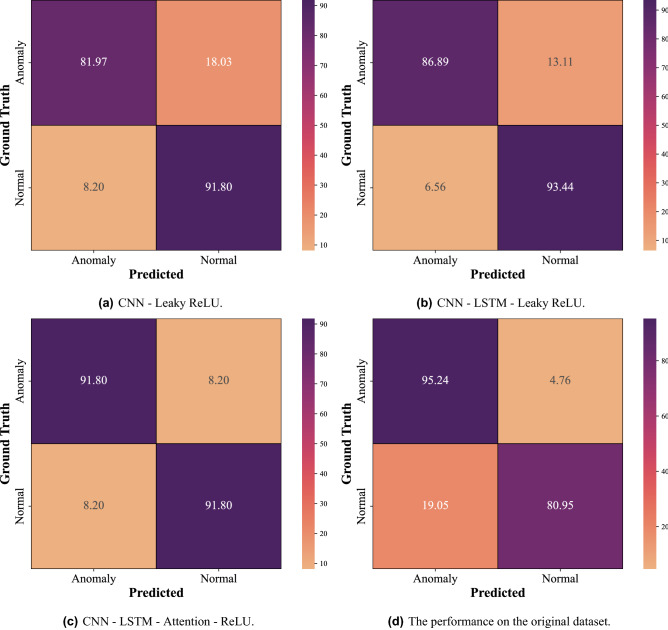
Comparison of different methods.

In CNN architecture, we run experiments with the Leaky ReLU activation function. The experiment parameters were identical to the CNN architecture in the proposed method. However, two fully connected layers we used at the end of CNN for classification. According to Table 4, the overall accuracy for this method was only 86.89%, which is lower than the accuracy of our proposed method (92.62%). Figure 6a shows the confusion matrix for this method. Table 5 shows precision, recall, F1-score for each class.

**Table 5:** The classification results of only CNN using Leaky ReLU.

	Precision	Recall	F1-score	No.
Anomaly	0.91	0.82	0.86	61
Normal	0.84	0.92	0.88	61
Accuracy			0.87	122
Macro avg	0.87	0.87	0.87	122
Weighted avg	0.87	0.87	0.87	122

No. indicates the number of samples.

##### CNN-LSTM-Leaky ReLU

This part experimented with CNN uses the Leaky ReLU activation function in conjunction with LSTM. This experiment tests whether incorporating an attention layer into the model is effective. In this method, accuracy achieved 90.16%, which is less than our proposed method (accuracy of 92.62%). It is clear that the accuracy of the model was improved by including the attention layer. In theory, with the attention layer, the LSTM is supposed to invest more computing power of that small but important part of the input, so the network enhances these parts and fades out the rest. The confusion matrix for this method is shown in Fig. 6b. Table 6 shows precision, recall, F1-score for each class.

**Table 6:** The classification results of CNN and LSTM using Leaky ReLU.

	Precision	Recall	F1-score	No.
Anomaly	0.93	0.87	0.90	61
Normal	0.88	0.93	0.90	61
Accuracy			0.90	122
Macro avg	0.90	0.90	0.90	122
Weighted avg	0.90	0.90	0.90	122

No. indicates the number of samples.

##### CNN-LSTM-Attention-ReLU

This part experimented with CNN architecture uses the ReLU activation function in conjunction with attention-based LSTM. The confusion matrix for this method is shown in Fig. 6c. In this experiment, the model was run with ReLU activation to prove it is less effective than Leaky ReLU activation on our dataset. When using the ReLU activation function, the accuracy was 91.80%, while using Leaky ReLU, the accuracy was higher (92.62%). As Leaky ReLU has a slope of 0.3 instead of 0, CNN can train faster and avoid the ‘dying ReLU’ problem on our dataset. Table 7 shows precision, recall, F1-score for each class.

**Table 7:** The classification results of CNN and attention-based LSTM using ReLU.

	Precision	Recall	F1-score	No.
Anomaly	0.92	0.92	0.92	61
Normal	0.92	0.92	0.92	61
Accuracy			0.92	122
Macro avg	0.92	0.92	0.92	122
Weighted avg	0.92	0.92	0.92	122

No. indicates the number of samples.

##### The performance on the original dataset

To test the efficiency of the data augmentation process, the proposed model in section 2 was run on both the original and augmented datasets. Table 8 shows precision, recall, F1-score for each class. The accuracy on the augmented dataset reached 92.62% whereas the accuracy on the original dataset only reached 88.10% (Table 9). The accuracy of our proposed method on the augmented dataset (402 sounds) is clearly higher than on the original dataset (201 sounds). The confusion matrix for our proposed method on the original dataset is shown in Fig. 6d.

**Table 8:** The classification results of CNN and attention-based LSTM using Leaky ReLU on the original drill dataset.

	Precision	Recall	F1-score	No.
Anomaly	0.83	0.95	0.89	21
Normal	0.94	0.81	0.87	21
Accuracy			0.88	42
Macro avg	0.89	0.88	0.88	42
Weighted avg	0.89	0.88	0.88	42

No. indicates the number of samples.

**Table 9:** The comparison of the original and augmented datasets using the same proposed method (CNN-LSTM-Attention-Leaky ReLU).

Dataset	Mean accuracy (%)
Augmented dataset (402 sounds)	92.62
Original dataset (201 sounds)	88.10

#### Discussion

The sound is too short, and the balanced dataset has too few samples, which are the two major challenges in developing a machine failure detection system for Valmet AB. To begin with, it is difficult to apply data augmentation methods to short sounds. Some modern data augmentation strategies, such as synthesizing new data using generative models, have recently attracted the interest of researchers. GAN, for example, is a common generative model used to synthesis new data from a small dataset in image processing and computer vision. The drilling sounds in Valmet’s dataset, on the other hand, are far too short to be used with state-of-the-art GAN. Furthermore, a model with too many parameters may underfit a limited training dataset. When deep learning models fail to catch the underlying trends in data, this is known as underfitting. As a result, the model will make numerous inaccurate predictions. To avoid underfitting, it is required to utilize a larger dataset with longer sounds. However, due to the high costs and labor-intensive nature of capturing and identifying sounds in factories, it is not feasible to collect large and balanced datasets. On a limited dataset, our proposed method can be utilized to develop a classification model. Drill sounds may be recorded and identified using this classification model right at the factory. A skilled technician can confirm the accuracy of the recorded sounds identified by this model. These new sounds are then added to a bigger dataset. When a deep learning model is trained on a larger dataset, it can generate better results.

### Results on Seibold’s dataset

**Figure 7:**
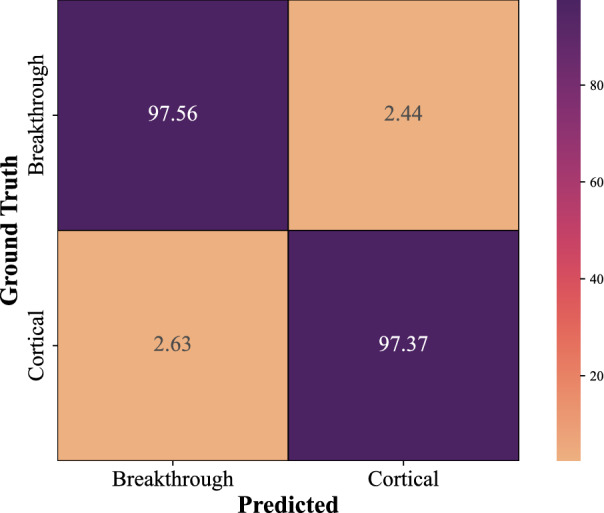
The confusion matrix for the proposed model (CNN-LSTM-Attention-Leaky ReLU) on Seibold’s dataset.

Our proposed method is validated on Seibold’s dataset to demonstrate its effectiveness as well as to avoid bias in the specified dataset from Valmet AB. Results of the experiment indicate that the proposed method achieves greater accuracy than the baseline system using ResNet-18^40^ in the previous study^26^ on the same dataset. As shown in Table 2, the mean accuracy of our proposed method on this dataset reached 97.47%, whereas, Seibold et al.^26^ reached an accuracy of 91.90%. Figure 7 depicts the confusion matrix using Seibold’s dataset with our proposed method, while the precision, recall, and F1-score for each class are shown in Table 10. According to these results, our proposed method is capable of efficiently processing short and small sound datasets, such as those of Valmet’s and Seibold’s datasets.

**Table 10:** CNN with Leaky ReLU in conjunction with attention-based LSTM on Seibold’s dataset.

	Precision	Recall	F1-score	No.
Breakthrough	0.98	0.98	0.98	41
Cortical	0.97	0.97	0.97	38
Accuracy			0.97	79
Macro avg	0.97	0.97	0.97	79
Weighted avg	0.97	0.97	0.97	79

No. indicates the number of samples.

### Results on UrbanSound8K

**Figure 8:**
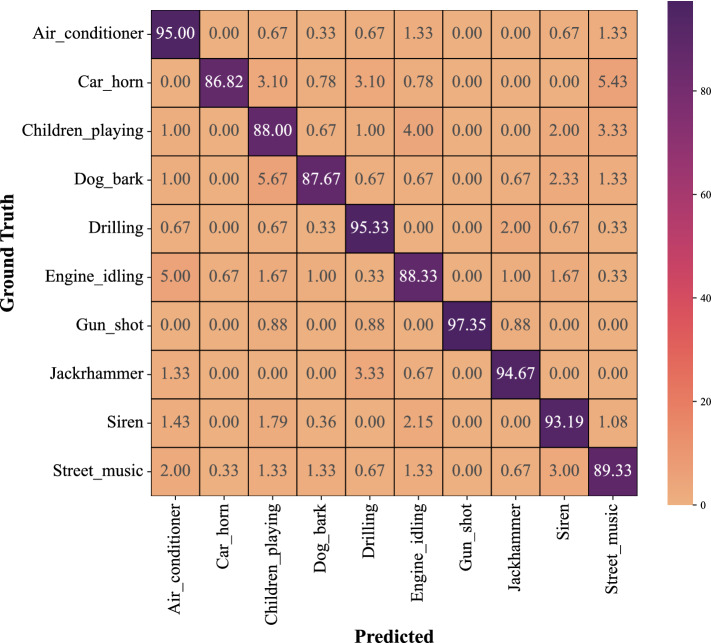
The confusion matrix for the proposed model (CNN-LSTM-Attention-Leaky ReLU) on UrbanSound8K dataset.

Figure 8 depicts the confusion matrix of the proposed method on the UrbandSound8K benchmark dataset. In Table 11, precision, recall, and F1-score for each class in the UrbandSound8K are presented. As shown in Table 2, our proposed method achieves better accuracy than state-of-the-art and the latest methods on the UrbandSound8K dataset. The mean accuracy of our proposed method is 91.45%, while they are 70.90%, 89.00%, and 90.07% for Stride-Ds-24^37^, 1D CNN^38^, and AudioCLIP^39^, respectively. These findings suggest that our method outperforms modern techniques on multi-class datasets with longer sounds. Additionally, it validates the generalization of our proposed method, which works well not only on small and short sound datasets but also on large datasets with many classes and long sounds.

**Table 11:** CNN with Leaky ReLU in conjunction with attention-based LSTM on UrbandSound8K dataset.

	Precision	Recall	F1-score	No.
Air_conditioner	0.89	0.95	0.92	300
Car_horn	0.97	0.87	0.92	129
Children_playing	0.87	0.88	0.87	300
Dog_bark	0.95	0.88	0.91	300
Drilling	0.92	0.95	0.94	300
Engine_idling	0.90	0.88	0.89	300
Gun_shot	1.00	0.97	0.99	113
Jackhammer	0.95	0.95	0.95	300
Siren	0.89	0.93	0.91	279
Street_music	0.90	0.89	0.90	300
Accuracy			0.91	2621
Macro avg	0.92	0.92	0.92	2621
Weighted avg	0.92	0.91	0.91	2621

No. indicates the number of samples.

## Conclusion

In this article, a deep learning model was proposed for a drill fault detection system. Besides, time-shifting and volume control augmentation methods were applied to increase the number of sounds in the small dataset. The sounds in the augmented dataset were converted into log-Mel spectrograms and were used to train the proposed CNN architecture with the Leaky ReLU activation function in conjunction with attention-based LSTM for detecting drill failure. It was found that the overall accuracy of our proposed system reached 92.62% on our Valmet’s dataset. In terms of identifying broken drill bits, the accuracy of the proposed method is acceptable. This method has a huge potential to be used to diagnose faults in industrial machines. It is a non-invasive method of diagnosing machine failure using short sounds or small datasets. Moreover, this paper used both a private dataset with small and short sounds namely Seibold’s dataset, and a benchmark UrbanSound8K dataset to validate the effectiveness and generalization of the proposed method. Studies show that our proposed method is more accurate than the state-of-the-art and the latest methods on both datasets. On the Seibold’s and UrbandSound8K datasets, our proposed method reaches an accuracy of 97.47% and 91.45%, respectively. We are considering combining sound and images to identify drilling errors and improve drilling error detection results in the future. Aspects of having many events in the same sound, such as polyphonic sounds, which blended both anomalous drill sounds and others, will also be examined.

## Data Availability

The datasets generated during and/or analysed during the current study are available from the corresponding author on reasonable request.
